# Combining chemotherapy and autologous peptide‐pulsed dendritic cells provides survival benefit in stage IV melanoma patients

**DOI:** 10.1111/ddg.14334

**Published:** 2020-11-16

**Authors:** Klaus Eisendle, Georg Weinlich, Susanne Ebner, Markus Forstner, Daniela Reider, Claudia Zelle‐Rieser, Christoph H. Tripp, Peter Fritsch, Patrizia Stoitzner, Nikolaus Romani, Van Anh Nguyen

**Affiliations:** ^1^ Department of Dermatology Venereology and Allergology Medical University of Innsbruck Innsbruck Austria; ^2^ Department of Dermatology and Venerology Central Hospital of Bolzano Italy; ^3^ Department of Visceral Transplant and Thoracic Surgery Medical University of Innsbruck Innsbruck Austria

## Abstract

**Background and Objectives:**

We examined retrospectively whether the combination of standard dacarbazine (DTIC) and/or fotemustine chemotherapy and autologous peptide‐loaded dendritic cell (DC) vaccination may improve survival of stage IV melanoma patients. Furthermore, a small cohort of long‐term survivors was studied in more detail.

**Patients and methods:**

Between 1998 and 2008, 41 patients were vaccinated at least three times with DCs while receiving chemotherapy and compared to all other 168 patients in our database who only received chemotherapy (1993–2008).

**Results:**

Median life expectancy of patients receiving additional DC‐vaccination was 18 months, compared to eleven months for patients under standard chemotherapy alone. In contrast to patients with other haplotypes, the HLA‐A1/A1 subset of DC‐treated patients showed significantly lower median survival (12 vs. 25 months). Autoantibodies were frequently detected in serum of both vaccinated and non‐vaccinated patients, and there was no correlation between titers, loss or appearance of autoantibodies and survival. Additionally, phenotyping of DCs and PBMCs also did not reveal any conspicuous correlation with survival.

**Conclusions:**

Combining standard chemotherapy and DC vaccination appears superior to chemotherapy alone. The impact of HLA haplotypes on survival emphasizes the importance of a careful selection of patients with specific, well‐defined HLA haplotypes for future vaccination trials using peptide‐pulsed DCs, possibly combined with checkpoint inhibitors.

## Introduction

Skin cancer, prominently including cutaneous melanoma, is rising in incidence and continues to pose a major medical challenge [1]. Prior to the introduction of modern checkpoint inhibitor therapy [2], treatment efficiency of metastatic stage IV melanoma was unsatisfactory with median survival times ranging from four to nine months. Dacarbazine (DTIC) and fotemustine were the only approved chemotherapies for disseminated melanoma, although none of these agents led to an improvement in overall survival [3, 4]. The high immunogenicity of melanoma, which can induce cytotoxic T lymphocyte (CTL)‐dependent spontaneous regression, prompted a number of different immunotherapeutic approaches to increase immunological responses [2, 5, 6]. In particular, dendritic cell (DC) vaccination showed promise for treating patients with advanced melanoma [7]. Indeed, small, initial trials using DC vaccines reported the regression of individual metastases [8, 9]. Although an initial, large, double‐blind trial reported DC vaccination responses comparable to chemotherapy in metastatic melanoma [10] (critically discussed in [11]), importantly, a recent, thorough 12‐year follow‐up of DC monotherapy‐treated patients revealed that survival periods were in the same range as with ipilimumab, but lacking major toxicity [11]. In spite of the recent decline in the number of DC therapy trials (concomitant with the advent of immune checkpoint therapy) there is continued interest and activity in DC‐based anti‐cancer immunization [12, 13, 14, 15], an important reason being that, despite impressive therapeutic success of immune checkpoint blockade, not all patients respond. The problem of resistance development also remains to be solved [16]. Therefore, further research efforts are needed. General agreement exists that combining different treatment strategies will be beneficial. Dendritic cells could be an important component of such combinatory approaches, not least because of the crucial role they play in checkpoint blockade immunotherapy, as described in recent reports [17, 18, 19].

The purpose of this retrospective study was therefore to elucidate whether one such combination, namely standard chemotherapy plus autologous tumor peptide‐loaded DC vaccination, could provide a survival benefit over standard chemotherapy alone in stage IV melanoma patients. In addition, the incidence, development and prognostic significance of autoimmunity were evaluated in DC‐vaccinated patients and compared with those treated with conventional chemotherapy alone. A limited number of long‐term survivors were studied in greater depth for indicative DC features associated with improved survival.

## Material and Methods

### Patients

All patients with stage IV melanoma who had been vaccinated with DCs at least three times since initiation of vaccination therapy in our department from October 1998 until May 2008 were included (n = 41) and compared to all other stage IV melanoma patients in our database who had been treated with standard chemotherapy (DTIC, 1000 mg/m^2^ i.v. in 4‐week intervals, and/or fotemustine, 100 mg/m^2^ i.v. weekly for 3 weeks, then every 4 weeks) from December 1993 (start of our database) to May 2008 (n = 168). One limitation of our study is, that no randomization of treatment arms was performed, therefore a selection bias cannot be ruled out. Selection of patients occurred according to their HLA status and was further limited by lab capacities.

Of note, none of these patients received checkpoint blockade immunotherapy. In addition, for some patients from this cohort DC treatment was continued until 2014, at most. Again, no checkpoint blockade immunotherapy was applied during this period of continued DC treatment. Most patients started out with DC monotherapy. Additional chemotherapy was only administered upon progression of the melanoma. Injections of DC and chemotherapy were never given simultaneously. Instead, whenever possible, a DC injection was placed exactly between two chemotherapy cycles, or alternatively, one day before chemotherapy.

A description of all methods can be found in Supplementary Materials.

## Results

### Patient characteristics

Patient demographics are listed in Table 1. All patients were of white/Caucasian background and had AJCC stage IV melanoma. We compared two patient cohorts, group 1 with 41 patients receiving standard chemotherapy plus tumor peptide‐pulsed DC vaccination and group 2 with 168 patients receiving DTIC and/or fotemustine only. Group 1 included 16 females and 25 males with a mean age of 59 years: group 2 contained 94 females and 74 males with a mean age of 65 years. Group 1 patients were HLA‐typed: 18 patients had at least one allele for HLA‐A1, 17 for HLA‐A2, 9 for HLA‐A3 and three for HLA‐24, whereas 12 patients were homozygous for HLA‐A1, 12 for HLA‐A2 and 6 for HLA‐A3 (Table S1; online Supporting Information).

**Table 1:** Patient characteristics

	Group 1: additional DC vaccination n (%)	Group 2: standard chemotherapy n (%)
Randomized (n = 209)	41 (100 %)	168 (100 %)
Caucasian	41 (100 %)	168 (100 %)
Median age (years)	59	65
*Sex*		
Male	25 (61 %)	74 (44 %)
Female	16 (39 %)	94 (56 %)
*AJCC M category*		
M1a	3 (7 %)	35 (21 %)
M1b	2 (5 %)	20 (12 %)
M1c	36 (88 %)	113 (67 %)

In a small, exploratory follow‐up cohort we further investigated three patients that survived under continuous DC therapy for 11 (57 vaccinations), 14 (59 vaccinations), and 14.5 (51 vaccinations; still alive in 2020) years and compared them to three patients that died within 14 months from the start of DC vaccinations.

### Survival of patients

**Figure 1:**
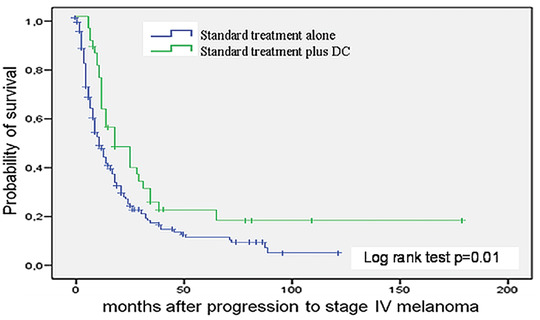
Kaplan‐Meier curves of overall survival by treatment arm. Arm A (in green: standard chemotherapy with DTIC and/or fotemustine), 168 patients and 136 deaths; arm B (in blue: standard chemotherapy plus DC vaccination), 41 patients and 31 deaths. *P* = 0.01 by log rank test. Censored observations are indicated by vertical bars.

**Figure 2:**
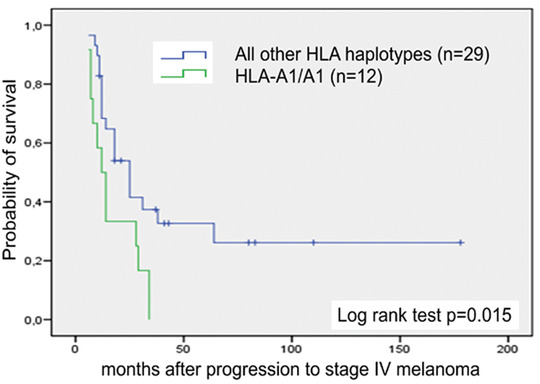
Kaplan‐Meier curves of overall survival of additionally DC‐vaccinated patients (group 1) by HLA haplotype. Arm A (in green: HLA‐A1/A1 haplotype), 12 patients and 12 deaths; arm B (in blue: all other HLA haplotypes), 29 patients and 19 deaths. *P* = 0.015 by log rank test. Censored observations are indicated by vertical bars.

The median overall survival of all stage IV melanoma patients within the observation period from 10/1998 to 05/2008 was 12 months (95 % confidence interval [CI]: 10.0–14.0) (Figure 1). Patients who were additionally vaccinated with autologous peptide‐pulsed DCs (group 1) had a median life expectancy of 18 months (95 % CI: 7.4–28.5), which was significantly higher than the eleven months – life expectancy of stage IV melanoma patients who received standard chemotherapy alone (group 2) (95 % CI: 8.5–13.5; log rank test *P* = 0.01) (Figure 1). Multivariate Cox regression analysis revealed a relative risk for worse outcome in male patients aged over 60 years (95 % CI: 0.6–1.2 and 0.8–1.5, respectively). As the unvaccinated patients were significantly older than the vaccinated ones we performed univariate and multivariate Cox regression analysis for survival, showing that the patients’ age did not impact the survival once having reached stage IV melanoma (Table S2; online Supporting Information). Importantly, the homozygous HLA‐A1/A1 subset of DC‐treated patients showed a significantly lower median survival of 12 months (95 % CI: 7.5–16.5) compared to patients carrying other haplotypes with a median survival of 25 months (95 % CI: 16.1–33.9; log rank test *P* = 0.015) (Figure 2).

### Development of autoimmunity: autoantibodies and vitiligo

**Figure 3:**
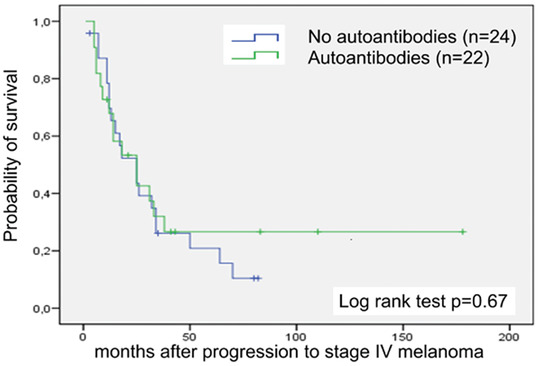
Kaplan‐Meier curves of overall survival by the presence of autoantibodies. Arm A (in green: patients with autoantibodies at second measurement), 22 patients and 15 deaths; arm B (in blue: patients without autoantibodies at second measurement), 24 patients and 20 deaths. *P* = 0.67 by log rank test. Censored observations are indicated by vertical bars.

The presence, new development and loss of autoantibodies within the ten years observation period from 10/1998 to 05/2008 are summarized in Table 2. We analyzed 25 patients from group 1 and 21 from group 2. From the date of diagnosis autoantibodies (one or more) were detected in 13 of 25 (52 %) DC‐vaccinated (group 1) and in 11 of 21 (52 %) chemotherapy‐only‐treated (group 2) stage IV melanoma patients (*P* = 0.7). Between the first and the second measurement (18 months) autoantibodies appeared in eight (32 %) DC‐vaccinated and four (19 %) chemotherapy‐treated patients (*P* = 0.2). A loss in autoantibodies was observed in three patients of each treatment arm (*P* = 0.6). Two patients treated with standard chemotherapy alone had vitiligo at the time of progression to stage IV, whereas two DC‐vaccinated patients developed vitiligo during treatment. Notably, the presence, new development and loss of autoantibodies were not associated with a survival benefit, neither within each treatment group nor when all patients were considered together (Figure 3).

**Table 2:** Presence, new development and loss of autoantibodies in stage IV melanoma patients receiving standard chemotherapy plus DC vaccination (group 1) or standard chemotherapy alone (group 2)

Autoantibodies	Group 1 (standard chemotherapy plus additional DC vaccination) (n = 25)	Group 2 (standard chemotherapy only) (n = 21)
First measurement	13 (53 %)	11 (52 %)
Second measurement	14 (56 %)	8 (36 %)
New development of autoantibodies	8 (32 %)	4 (21 %)
Loss of autoantibodies	3 (12 %)	3 (14 %)

### Influence of dendritic cell quality on clinical outcome

Populations of peptide‐loaded mature DCs administered to the patients were routinely monitored and found to be of high quality as described in detail in Supplementary Materials.

### Immunological follow‐up of a limited number of long‐term surviving patients

*Immunohistochemistry:* PD‐L1 expression on tumor cells is widely used as an – albeit imperfect – prognostic marker for responsiveness to immune checkpoint inhibitor therapy [20, 21]. Similarly, we hypothesized that high PD‐L1 expression on tumor cells may correlate with short survival, and *vice versa*. A small, exploratory sample of pre‐DC treatment tumors from five short‐term (< 2 years) and three long‐term (> 11 years) survivors was immunohistochemically probed for PD‐L1. Results were not conclusive. Further studies with larger samples are needed.

*Flow cytometry:* Cryopreserved peripheral blood mononuclear cells (PBMCs) from time points of the first leukapheresis, that is, before the start of DC vaccinations (for 3 short and 2 long‐term survivors) and, in addition, from 15, 55 and 82 months after the start of DC therapy for either of the three the long‐term survivors were analyzed with large panels of antibodies (Table S3, online Supplement Information). Peripheral blood mononuclear cells of patients before treatment showed no differences in the relative proportions of lymphoid cell populations (CD4^+^, CD8^+^ T cells and total CD56^+^CD3^–^ NK cells) (Figure S1, online Supplement Information). Percentages of activated T cells, as determined by HLA‐DR staining, were also similar between the two groups (data not shown). Likewise, proportions of myeloid cell types (CD14^+^ monocytic cells, CD11c^+^CD1c^–^CD141^+^ cDC1 and CD11c^+^CD1c^+^ cDC2) did not differ grossly between long‐term and short‐term survivors (Figure S2, online Supplement Information). Percentages of activated CD14^+^ cells and cDC2 (as determined by CD83 expression) were also unchanged, and so was the expression of PD‐L1 on DCs (data not shown), the latter being a DC feature that, when experimentally blocked, was found to enhance T‐cell responses [22]. For long term survivors, percentages of lymphoid and myeloid cells were unaltered during treatment (data not shown).

Regarding immune effector cells, it is noteworthy that NK cells may have been critical in one of the patients (14 yrs. survival) who had markedly elevated numbers of CD56^+^ NK cells in the blood throughout the course of therapy. This, however, was already evident before the start of DC vaccination and can therefore not be ascribed to the DC therapy. The other long‐term survivors did not show such conspicuously and persistently elevated NK levels. Nevertheless, one observation might provide a “lead” to further analyses on subsets of NK cells, namely the CD56‐bright, cytolytically potent minority of NK cells in the blood versus the majority of CD56‐dim NK cells [23]. In our small cohort the long (> 10 years) surviving patients had proportionally slightly more CD56‐bright NK cells in their blood as compared to the short (< 2 years) surviving patients (ratios of CD56‐bright to CD56‐dim: 0.36 for long and 0.10 for short term survivors) (Figure S3, online Supplement Information).

## Discussion

The principal finding of this retrospective study was that in contrast to the standard chemotherapy group, patients survived significantly longer under additional DC vaccination. The median life expectancy of patients who received additional DC‐vaccination was 18 months, compared to 11 months in patients who were treated with standard chemotherapy alone. Of note, these data are from the period from 1998 to 2008, and therefore none of the patients had received immune checkpoint immunotherapy or kinase inhibitors. Although DC vaccination can induce antigen specific immune responses in melanoma patients, only a few trials have shown a correlation between survival and detectable immune responses [24]. Numerous reasons were proposed for this discordance, ranging from the wide variety of methods for generating DCs to the routes of DC administration. Livingston et al. [25] and Wallack et al. [26], for example, found statistically significant survival benefits in the active specific immunotherapy group. Likewise, Kyte et al. [27] reported that DC‐vaccinated patients experienced a prolonged overall survival when retrospectively compared to a control group of non‐vaccinated patients receiving standard treatment. Although the first large‐scale, comparative clinical study by Schadendorf et al. [10] failed to demonstrate that DC‐vaccination was superior to DTIC treatment, explorative subgroup analyses indicated that HLA‐A2^+^/HLA‐B44^–^ vaccinated patients survived longer than DTIC treated patients. This difference was not observed in the DTIC arm. Similarly, Sosman et al. [28] documented a similarly beneficial effect of an allogeneic tumor vaccine in patients positive for HLA‐A2.

Our findings are consistent with these data, and with recent observations by Gross et al. [11], who found an association of HLA‐A1/A1 and unfavorable clinical outcome, with other haplotypes being more frequent in long‐term survivors. One of several possible reasons for the better response of HLA‐A2 patients may be that more peptides on more DC batches were administered per vaccination for the A2 haplotype. Based on these observations, we strongly suggest that the expression pattern of HLA haplotypes could have a major impact on vaccination efficacy. Therefore, we recommend that only patients with specific, well‐defined HLA haplotype characteristics should be enrolled in future vaccination trials using peptide‐loaded DCs.

There is increasing evidence for a potential synergistic effect of DC vaccination and conventional chemotherapy [29, 30, 31, 32]. The development of drug resistance to conventional chemotherapy is considered to be the major cause for treatment failure in many types of cancer. One way in which cancer cells become resistant is through increased expression of regulator proteins of apoptosis (e.g., survivin). In a combination scenario, chemotherapy would kill most cancer cells, leaving behind only resistant cells expressing high levels of these proteins. This would render them particularly susceptible to killing by DC vaccination‐induced T cells (e.g., against survivin) [31, 33].

In addition, immunogenic cell death may be brought about by certain forms of chemotherapy, which may provide copious tumor antigen for DCs to present to T cells [34, 35, 36]. The improved efficacy of DC vaccination might also be due to the depletion of CD4^+^, CD25^+^, Foxp3^+^ regulatory T cells (Treg) by the administration of chemotherapy prior to immunotherapy [37]. These cells were shown to play an inhibitory role in anti‐tumor responses.

As has been shown by others, our retrospective study revealed that vaccination with autologous tumor peptide‐pulsed DCs is a safe procedure and does not induce more autoimmune phenomena [8, 9, 38] as compared to the control chemotherapy treatment. This contrasts with the well‐known occurrence of autoantibodies [39] and autoimmune pathology [40, 41] in immune checkpoint immunotherapy. In general, autoantibodies were frequently detected in both vaccinated and non‐vaccinated stage IV melanoma patients. Also, vitiligo occurred in only two patients of each treatment group. It should be noted that there was no correlation between development of autoimmunity and a better prognosis, strongly indicating that the survival benefit seen in vaccinated stage IV melanoma patients was due to the induction of specific antitumor immunity.

In addition to mere efficacy considerations, but also in view of our autoantibody findings, it should be emphasized that DC‐based immunotherapy is well tolerated. We observed this in our patients over the years, and it could also be objectified in quality of life (QoL) studies in melanoma [42] and – much earlier – in renal cell carcinoma [43].

In search for possible immune correlates for prolonged survival we followed up a small, exploratory cohort of DC plus chemo‐treated stage IV metastatic melanoma patients representing the extremes of the observed spectrum, that is, patients who survived for more than eleven years versus patients who died within 14 months of the onset of DC therapy. PD‐L1 expression patterns on the primary melanomas of five short and three long‐term survivors were not conclusive. More samples are needed to reveal a possible correlation. Nor did the flow cytometry analysis of lymphoid and myeloid leukocytes subsets in pre‐therapy PBMCs, including DC subsets (cDC1, as defined by CD141 and cDC2) of three short and two long‐term survivors provide a strong and reliable prognostic marker for responsiveness to DC therapy, specifically survival. The only possible “lead” was found in NK cells of three short and three long‐term survivors, where long‐term survivors had proportionally more CD56‐bright, strongly cytotoxic NK cells [23]. This observation, however, needs more investigation. Interestingly, the recent 12‐years follow‐up analysis of DC‐vaccinated melanoma patients by the Erlangen group [11] could not corroborate some correlations that would have seemed obvious at first glance: Prolonged survival did not correlate with numbers of regulatory T cells or myeloid‐derived suppressor cells (MDSC) cells, or with frequencies of vaccine‐specific T cells (as determined by Elispot and tetramer staining) in the blood, but rather with strong reactions at the vaccine injection sites, and with blood eosinophilia of 20 % or more at any time point during (though not before) vaccination therapy [44]. Similarly, a substantial proportion (50 %) of our patients treated with DC‐based immunotherapy plus chemotherapy experienced at least once an eosinophilia of 5 % or more during the course of therapy, but only one of 41 (2.4 %) reached levels of more than 10 %, and none had more than 20 %. However, a trend, although not statistically significant, appeared. Eosinophilia of 5–10 % occurred in eleven of 14 (79 %) long‐term surviving patients (> 18 months) as compared to 10 of 28 (36 %) patients surviving less than 18 months. The reason for the absence of eosinophilias beyond 20 % remains elusive. Speculatively, the concomitant chemotherapy might have had an influence. Local reactions in response to the vaccinations were occasional reddening of the injection site and occasional sub‐febrile temperature. However, these observations were not systematically correlated with clinical responses. An elevated ratio of myeloid to lymphoid (T, B, NK) cells in the blood was reported to correlate with poor survival and low levels of PEBP1 expression [45]. Our low sample size did not allow substantiation of this observation. Interestingly, patient survival also did not correlate with the phenotypical quality of administered mature DCs. This observation emphasizes, however, that monocyte‐derived DCs generated from the blood of cancer patients can mature perfectly well and are fully intact.

In summary, we showed herein that standard chemotherapy in combination with autologous tumor peptide‐loaded DC vaccination is more effective in stage IV melanoma patients than standard chemotherapy alone. Moreover, the response to DC vaccination appears to be influenced by the HLA haplotype. In this respect, combining DC vaccination with treatment regimens other than chemotherapy, such as immune checkpoint inhibitors and inhibitors of the RAS/RAF/MEK/ERK pathway, may be worthy of further consideration to circumvent the complex tumor escape mechanisms and thus to achieve better clinical responses. Indeed, some initial trials have already been performed, albeit with limited success [14, 46]. This underscores the need for further optimization and standardization of current DC vaccination protocols, especially in terms of DC generation, administration (e.g., targeting of antigen to DC subsets [47, 48, 49, 50]) and antigen preparation. DC will remain an important component of the (immuno)therapeutic “toolbox” against cancer.

## Funding and Acknowledgement

Personnel (S.E., D.R., M.F.) and infrastructure for the manufacturing and quality control of DC for therapy was financed over the years by Kompetenzzentrum Medizin Tirol and the Oncotyrol research consortium. Oncotyrol was funded by the Austrian Research Promotion Agency (FFG) and the Regional Government (Standortagentur Tirol). We highly appreciate the continuous participation of *tirol‐kliniken*, who served as a partner of Oncotyrol. Additional support came from Austrian Science Fund projects P29919 to N.R. and P27001 to P.S.

This paper is dedicated to Prof. Peter Fritsch on the occasion of his 80^th^ birthday. He was Director of the Department of Dermatology & Venereology from 1981 to 2008. Under his auspices and with his input and support the largest part of the reported studies was performed.

## Conflict of interest

None.

## Supporting information

Supplement Information

Supplement Information

Table S1

Table S2

Table S3

Table S4

Figure S1

Figure S2

Figure S3
