# Supplementary material for: Combining chemotherapy and autologous peptide‐pulsed dendritic cells provides survival benefit in stage IV melanoma patients
Source: J Dtsch Dermatol Ges. 2020 Nov 16;18(11):1270–7. doi: 10.1111/ddg.14334 (PMC7756560; doi:10.1111/ddg.14334)
Supplement: Supplementary file 1 — Supplement Information [file DDG-18-1270-s001.docx]

Please note: Decimal points (.) should be changed to decimal commas (,) in the German version.

| Supplementary Fig. 1 | Ergänzende Abbildung 1 |
| --- | --- |
| % lymphoid cells | % lymphoide Zellen |
| cells | Zellen |
| long term | Langzeitüberlebende |
| short term | Kurzzeitüberlebende |

| Supplementary Fig. 2 | Ergänzende Abbildung 2 |
| --- | --- |
| % CD14^+^ cells | % CD14^+^-Zellen |
| % dendritic cells | % dendritische Zellen |
| cells | Zellen |
| long term | Langzeitüberlebende |
| short term | Kurzzeitüberlebende |

| Supplementary Fig. 3 | Ergänzende Abbildung 3 |
| --- | --- |
| percent positive cells | Prozentsatz positiver Zellen |
| patient (lower case) | Patient (upper case) |
| long term survivors | Langzeitüberlebende |
| short term survivors | Kurzzeitüberlebende |
| low during | niedrig während |
| low before | niedrig vor |
| high during | hoch während |
| high before | hoch vor |
