# Supplementary material for: Combining chemotherapy and autologous peptide‐pulsed dendritic cells provides survival benefit in stage IV melanoma patients
Source: J Dtsch Dermatol Ges. 2020 Nov 16;18(11):1270–7. doi: 10.1111/ddg.14334 (PMC7756560; doi:10.1111/ddg.14334)
Supplement: Supplementary file 2 — Supplement Information [file DDG-18-1270-s002.docx]

[[Online Supplement Information]]

**Combining chemotherapy and autologous peptide-pulsed dendritic cells provides survival benefit in stage IV melanoma patients**

Klaus Eisendle^1, 2^, Georg Weinlich^1^, Susanne Ebner^1, 3^, Markus Forstner^1^, Daniela Reider^1^, Claudia Zelle-Rieser^1^, Christoph H. Tripp^1^, Peter Fritsch^1^, Patrizia Stoitzner^1^, Nikolaus Romani^1^, Van Anh Nguyen^1^

(1) Department of Dermatology, Venereology and Allergology, Medical University of Innsbruck, Innsbruck, Austria

(2) Department of Dermatology and Venerology, Central Hospital of Bolzano, Italy

(3) Department of Visceral, Transplant and Thoracic Surgery, Medical University of Innsbruck, Innsbruck, Austria

**Short title:** Combining dendritic cells and chemotherapy in melanoma

Correspondence to

Van Anh Nguyen, MD

Department of Dermatology, Venereology & Allergology

Medical University of Innsbruck

Anichstrasse 35

6020 Innsbruck, Austria

E-mail: van.nguyen@i-med.ac.at

**Supplementary Materials And Methods**

*Patients*

Ethical approval (AN821/1998; UN1100/2000; UN1597/2003) was given by the Ethical Committee of the Medical University of Innsbruck for patients, that had originally been included in already published clinical trials [1, 2] and for the analysis of cells and data from those patients whose treatment with DCs was continued on a compassionate use basis (AN2016-0130).

*DC generation, vaccine preparation and peptides used*

Peripheral blood mononuclear cells (PBMCs) were harvested from leukapheresis by standard density centrifugation (Lymphoprep; 1.077 g/ml; Axa-Shield) and processed immediately and/or frozen in aliquots until use. In order to increase numbers of myeloid cells in the blood, patients were treated one or two days before leukapheresis with 300 µg of human G-CSF (granulocyte colony-stimulating factor/CSF-3, Neupogen^TM^; Hoffmann-LaRoche, Basel, Switzerland). To generate monocyte-derived DCs, essentially as described [2–4], PBMCs were plated onto plastic flasks (NUNC Cell Factories^TM^, NUNC-Thermofisher) and allowed to adhere for 1 h at 37 °C in 5 % CO_2_. Non-adherent cells were removed, and the remaining adherent monocyte-rich fractions were cultured in RPMI 1640 (Bio-Whittaker/Lonza, Walkersville, MD, USA), 0.4 % refobacin (Merck Serono, Darmstadt, Germany), 1 % L-glutamin (Lonza, Basel, Switzerland), 1 % heat-inactivated autologous plasma, 800 U/ml GM-CSF (Leukine^TM^; sargramostim, Bayer, Berlin, Germany; specific activity 5.6 x 10^6^IU/mg) and 500 U/ml IL-4 (CellGro^TM^, Cellgenix, Freiburg, Germany; specific activity 0.8–1.4 x 10^7^ IU/mg) for 6 days. To mature DCs from day 6 to 7, cells were stimulated with a cytokine cocktail, first described by Jonuleit et al. [5] consisting of 600 U/ml TNF-α (10 ng/ml from Cellgenix), 1.000 U/ml IL-1β (2 ng/ml from Cellgenix), 1.000 U/ml IL-6 (Cellgenix), and 1 µg/ml prostaglandin E2 (Minprostin; Pfizer, Karlsruhe, Germany). All cell culture reagents and cytokines were of GMP quality.

For immunization, 7-Day DCs were harvested, and their viability (by trypan blue exclusion), morphology (by phase contrast) and phenotype were characterized by flow cytometry using a panel of monoclonal antibodies (mAbs) against HLA-DR (BD Pharmingen, San Jose, CA, USA), CCR7 (R&D, Minneapolis, MN, USA) and CD3, CD14, CD19, CD25, CD40, CD56, CD83, CD86 (BD Pharmingen). These mature DCs were then pulsed with MHC class I- and II-restricted peptides at 6.25 µg/ml and 3.75 µg/ml, respectively, for 3 h. The MHC class I- and II-restricted peptides (Bachem/Clinalfa, Bubendorf/Läufelfingen, Switzerland) are listed in Table S4 (online Supplement information). In later phases of the study batches of peptide-loaded mature DCs were frozen and thawed prior to vaccination [6]. Subsequently, peptide-loaded DCs were collected, washed and administered at a concentration of 4 x 10^6^ DCs in 1 ml PBS and 1 % HSA (human serum albumin; Biotest Pharma GmBH, Dreieich, Germany) by intradermal injection in the armpits or groins in the close proximity of lymph nodes. Vaccination intervals were two weeks for the first three vaccinations and every four weeks thereafter. Samples of vaccine were tested for bacterial and fungal sterility before patient administration. Long-term survivors were vaccinated on a compassionate use basis in intervals up to four times a year.

*Assessment of autoimmunity*

To detect any serological autoimmune reaction, anti-nuclear (ANA; titers being 1 : 80 or above were considered positive), anti-DNA, anti-cardioliopin (ACA) and anti-thyroid autoantibodies were determined in 25 vaccinated stage IV melanoma patients and in 21 matched chemotherapy treated stage IV melanoma patients prior to start of therapy and after 18 months. Apart from this, patients’ records were examined for evidence or development of vitiligo.

*Immunohistochemistry*

Sections of paraffin-embedded tumor material of few selected patients were stained with monoclonal antibodies against PD-L1 (FDA-approved clone 22C3 Dako-Agilent) and PD-1 (clone NAT105, Biocare Medicale, Pacheco, CA, USA; cat.no. AC1 313/ AK) on the Dako-Omnis platform.

***Flow cytometry***

PBMC of selected patients, cryopreserved in the gaseous phase of liquid nitrogen, were thawed and stained with panels of fluorochrome-conjugated monoclonal antibodies (Table S3, online Supplement Information) aimed at possible therapy-related or pre-existing alterations in the proportions of myeloid and lymphoid blood leukocytes. Data were acquired on a FACS-Canto instrument and further analyzed by means of FloJo^TM^ software (Becton Dickinson).

*Statistical analysis*

Calculations were performed using SPSS statistical software (version 15.0; SPSS Inc., Chicago, IL, USA). Statistical correlations were assessed by the two-tailed Fisher’s exact test and the Mantel-Haenszel test. Kaplan-Meier curves were used to estimate the probabilities of survival and the log-rank test was used to compare time-to-event distributions. Progression was expressed by 95 % confidence intervals (CIs) and survival was tested by Cox regression analysis, assuming proportional hazards in a univariate as well as multivariate approach. p of < 0.05 was considered statistically significant.

**Supplementary Results**

*Influence of dendritic cell quality on clinical outcome*

Populations of peptide-loaded mature DCs administered to the patients had the following phenotypical characteristics in terms of percentages of marker-expressing cells: CD40 (93.12; range 100–34; n = 349), CD86 (98.34; range 100–60; n = 413), CD83 (88.13; range 100–44; n = 416), CD25 (65.68; range 95–13; n = 380), and low level CD14 (12.33; range 79–1; n = 414). These cumulative values represent the mean of the above indicated DC vaccination batches derived from a total of 40 leukapheresis, i.e., at least 40 separate DC generation processes, from 15 individual patients. Each leukapheresis yielded mature DCs for several vaccination batches that were cryopreserved and thawed on the day of vaccination. Importantly, mean fluorescence intensities for CD14 on DCs were at least one log lower than on monocytes in the freshly isolated PBMCs. The percentage of CD14-bright cells, i.e., like monocytes in the blood, was consistently below 5 %. Moreover, both immature and mature DC were largely non-adherent to tissue culture plastic. Furthermore, when mature DCs were cultured for another two days in the absence of cytokines (wash-out stability test [6]) they consistently remained non-adherent and viable and did not lose their phenotypical maturation markers. T cell stimulatory capacity of mature DCs in the allogeneic mixed leukocyte reaction was also consistently high. The quality of DCs in terms of maturation, i.e., levels of CD83, CD80, CD86 and CD25, did not positively correlate with patient survival, nor did the residual low levels of CD14 correlate negatively, as statistically assessed on the large number of samples mentioned above. In a limited number of patients' DC samples we additionally determined levels of CCR7 and TSLP-receptors (n = 60 and 30, respectively). This sample did not allow a correlation of these expression levels (73.57 ± 15.20 %, range 31–96 % for CCR7 and 84.65 ± 10.74 %, range 52–97 % for TSLP-R) to clinical parameters, though.

Supplementary References

1. Thurner B, Haendle I, Röder C et al. Vaccination with mage-3A1 peptide-pulsed mature, monocyte-derived dendritic cells expands specific cytotoxic T cells and induces regression of some metastases in advanced stage IV melanoma. J Exp Med 1999;190: 1669–78.
2. Schuler-Thurner B, Schultz ES, Berger TG et al. Rapid induction of tumor-specific type 1 T helper cells in metastatic melanoma patients by vaccination with mature, cryopreserved, peptide-loaded monocyte-derived dendritic cells. J Exp Med 2002;195: 1279–88.
3. Buschow SI, Ramazzotti M, Reinieren-Beeren IMJ et al. Survival of metastatic melanoma patients after dendritic cell vaccination correlates with expression of leukocyte phosphatidylethanolamine-binding protein 1/Raf kinase inhibitory protein. Oncotarget 2017; 8: 67439–56.
4. Gross S, Erdmann M, Haendle I et al. Twelve-year survival and immune correlates in dendritic cell-vaccinated melanoma patients. JCI Insight 2017; 2: e91438.
5. Jonuleit H, Kuhn U, Muller G et al. Pro-inflammatory cytokines and prostaglandins induce maturation of potent immunostimulatory dendritic cells under fetal calf serum-free conditions. Eur J Immunol 1997; 27: 3135–42.
6. Feuerstein B, Berger TG, Maczek C et al. A method for the production of cryopreserved aliquots of antigen-preloaded, mature dendritic cells ready for clinical use. J Immunol Methods 2000; 245: 15–29.

**Legends For Supplementary Figures And Tables**

Table S1 HLA types of vaccinated patients.

Table S2 Multivariate Cox regression analysis for survival.

Table S3 Antibodies used for phenotyping thawed PBMC from selected patients.

Table S4 Peptides used for loading dendritic cells for vaccinations.

Figure S1 Percentages of lymphoid cells were similar between long term and short term survivors: PBMCs were analyzed by flow cytometry for the presence of CD4^+^ and CD8^+^ T cells as well as CD3^–^CD56^+^ NK cells. Percentages are shown in all viable PBMCs for 2–3 patients of each group before treatment.

Figure S2 Percentages of myeloid cells were similar between long term and short term survivors: PBMCs were analyzed by flow cytometry for the presence of CD14^+^ cells as well as CD1c^+^ cDC2 and CD141^+^ cDC1. Percentages are shown in all viable PBMCs for 2–3 patients of each group before treatment.

Figure S3 Levels of CD56 on NK cells in PBMC. PBMCs were analyzed by flow cytometry for the expression levels of CD56 on CD3^–^CD56^+^ NK cells. Percentages are shown in all viable PBMCs for 2–3 patients of each group before and for long term patients also during treatment. (*Top panels*) Representative example of CD56 staining, highlighting the bright and dim subsets. (*Bottom panels*) Proportions of high and low CD56 expressing NK cells. CD56-high in blue; CD56-dim in red. Note that for short term survivors this analysis was performed only before the start of DC vaccinations. PBMCs of long term survivors during treatment were from 55, 82 and 15 months for patients 4–6, respectively.
