# Supplementary material for: Combining chemotherapy and autologous peptide‐pulsed dendritic cells provides survival benefit in stage IV melanoma patients
Source: J Dtsch Dermatol Ges. 2020 Nov 16;18(11):1270–7. doi: 10.1111/ddg.14334 (PMC7756560; doi:10.1111/ddg.14334)
Supplement: Supplementary file 3 — Table S1 [file DDG-18-1270-s003.docx]

Table S1 HLA types of vaccinated patients.

| **HLA* type** | **At least one allele**  n (%) | **Homozygous**  n (%) |
| --- | --- | --- |
| A1+ | 18 (43.9 %) | 12 (29.3 %) |
| A2+ | 17 (41.4 %) | 12 (29.3 %) |
| A3+ | 9 (21.9 %) | 6 (14.6 %) |
| A24+ | 3 (7.3 %) | 0 |
