# Supplementary material for: Combining chemotherapy and autologous peptide‐pulsed dendritic cells provides survival benefit in stage IV melanoma patients
Source: J Dtsch Dermatol Ges. 2020 Nov 16;18(11):1270–7. doi: 10.1111/ddg.14334 (PMC7756560; doi:10.1111/ddg.14334)
Supplement: Supplementary file 4 — Table S2 [file DDG-18-1270-s004.docx]

Table S2 Multivariate Cox regression analysis for survival.

|  | **Relative risk** | **95 % CI** | **p-value** |
| --- | --- | --- | --- |
| Age > 60 years (median 61.5) | 1.13 | 0.8-1.5 | 0.433 |
| Male sex | 0.85 | 0.6-1.2 | 0.310 |
| AJCC M category |  |  |  |
| M1a | 1 |  |  |
| M1b | 1.7 | 0.8-2.9 | 0.163 |
| M1c | 1.8 | 1.1-2.6 | 0.025 |
| Plus vaccination | 0.59 | 0.4-0.9 | 0.008 |
| Age as continuous variable (univariate) | 1.004 | 0.99-1.02 | 0.51 |
