# Supplementary material for: Combining chemotherapy and autologous peptide‐pulsed dendritic cells provides survival benefit in stage IV melanoma patients
Source: J Dtsch Dermatol Ges. 2020 Nov 16;18(11):1270–7. doi: 10.1111/ddg.14334 (PMC7756560; doi:10.1111/ddg.14334)
Supplement: Supplementary file 5 — Table S3 [file DDG-18-1270-s005.docx]

Table S3 Antibodies used for phenotyping thawed PBMC from selected patients.

|  | | | | | | | | |
| --- | --- | --- | --- | --- | --- | --- | --- | --- |
| *Panel 1, for the detection and characterization of lymphocytes in the blood* | | | | | | | | |
| *Antigen* | CD56 | / | CD4 | HLA-DR | CD3 | eFluor  L/D | CD8 | PD-1 |
| Fluorochrome | FITC | PE | PerCP-Cy5.5 | PE-Cy7 | APC | APC-Cy7 | BV421/Vio Blue | BV510/  AmCyan |
| Clone | NCAM16.2 |  | SK3 | L243 | UCHT1 |  | RPA-T8 | EH12.2H7 |
| Isotype | Mouse IgG2b, k |  | Mouse IgG1, k | Mouse IgG2a, k | Mouse IgG1, k |  | Mouse IgG1, k | Mouse IgG1, k |
| Source | BD |  | BD | Biolegend | Biolegend |  | Biolegend | Biolegend |

| *Panel 2, for the detection and characterization of dendritic cells in the blood* | | | | | | | | |
| --- | --- | --- | --- | --- | --- | --- | --- | --- |
| *Antigen* | CD83 | CD14 | CD1c | HLA-DR | PD-L2 | eFluor  L/D | PD-L1 | CD11c |
| Fluorochrome | FITC | PE | PerCP-Cy5.5 | PE-Cy7 | APC | APC-Cy7 | BV421/Vio Blue | BV510/  AmCyan |
| Clone | HB15e | HCD14 | L161 | L243 | MIH18 |  | MIH1 | B-ly6 |
| Isotype | Mouse IgG1, k | Mouse IgG1, k | Mouse IgG1, k | Mouse IgG2a, k | Mouse IgG1, k |  | Mouse IgG1, k | Mouse IgG1,k |
| Source | BD | Biolegend | Biolegend | Biolegend | BD |  | BD | BD |

| *Panel 3, for the detection and characterization of dendritic cells in the blood* | | | | | | | | | | | | | | | |  |
| --- | --- | --- | --- | --- | --- | --- | --- | --- | --- | --- | --- | --- | --- | --- | --- | --- |
| *Antigen* | CD14 | CD303 | | CD1c | | HLA-DR | | CD141 | | eFluor  L/D | | | CD163 | | CD11c | |
| Fluorochrome | FITC | PE | | PerCP-Cy5.5 | | PE-Cy7 | | APC | | APC-Cy7 | | | BV421/Vio Blue | | BV510/  AmCyan | |
| Clone | M5E2 | | AC144 | | L161 | | L243 | | AD5-14H12 | |  | GHI/61 | | B-ly6 | |  |
| Isotype | Mouse IgG2a, k | | Mouse IgG1, k | | Mouse IgG1, k | | Mouse IgG2a, k | | Mouse IgG1, k | |  | Mouse IgG1, k | | Mouse IgG1,k | |  |
| Source | Biolegend | | MB | | Biolegend | | Biolegend | | MB | |  | Biolegend | | BD | |  |

*Abbr.:* L/D, live/dead marker; BD, Becton-Dickinson; MB, Miltenyi Biotec; all antibodies were used at the concentrations / dilutions indicated by the manufacturers.
