# Supplementary material for: Combining chemotherapy and autologous peptide‐pulsed dendritic cells provides survival benefit in stage IV melanoma patients
Source: J Dtsch Dermatol Ges. 2020 Nov 16;18(11):1270–7. doi: 10.1111/ddg.14334 (PMC7756560; doi:10.1111/ddg.14334)
Supplement: Supplementary file 6 — Table S4 [file DDG-18-1270-s006.docx]

##### Table S4 Peptides used for loading dendritic cells for vaccinations.

| Peptide Name | HLA  restriction |  | C |  |  |  | Sequence | | | | | | | |  |  |  | N | Source |
| --- | --- | --- | --- | --- | --- | --- | --- | --- | --- | --- | --- | --- | --- | --- | --- | --- | --- | --- | --- |
|  | **HLA-A2** |  | 1 | 2 | 3 | 4 | 5 | 6 | 7 | 8 | 9 | 10 |  |  |  |  |  |  | (Product #) |
| Influenza Matrix | A2.1 |  | G | I | L | G | F | V | F | T | L |  |  |  |  |  |  |  | C-S-029 |
| Influenza B NP 85-94 | A2.1 |  | K | L | G | E | F | Y | N | Q | M | M |  |  |  |  |  |  | C-S-065 |
| Mage-3A2 271-279 | A2.1 |  | F | L | W | G | P | R | A | L | V |  |  |  |  |  |  |  | C-S-028 |
| Mart 1/Melan A, aa26-35 analogue | A2.1 |  | E | L | A | G | I | G | I | L | T | V |  |  |  |  |  |  | C-S-039 |
| gp 100, aa 209-217 ana* | A2.1 |  | I | M | D | Q | V | P | F | S | V |  |  |  |  |  |  |  | C-S-040 |
| Tyrosinase aa 368-376 | A2.1 |  | Y | M | D | G | T | M | S | Q | V |  |  |  |  |  |  |  | C-S-049 |
| GnTV nt 38-64 | A2.1 |  | V | L | P | D | V | F | I | R | C | V |  |  |  |  |  |  | C-S-050 |
| Mage-A10 aa254-262 | A2.1 |  | G | L | Y | D | G | M | E | H | L |  |  |  |  |  |  |  | C-S-053 |
| Mage-A4 aa 230-239 | A2.1 |  | G | V | Y | D | G | R | E | H | T | V |  |  |  |  |  |  | C-S-052 |
|  | **HLA-A1** |  | 1 | 2 | 3 | 4 | 5 | 6 | 7 | 8 | 9 |  |  |  |  |  |  |  | Quelle |
| Influenza NP | A1 |  | C | T | E | L | K | L | S | D | Y |  |  |  |  |  |  |  | C-S-042 |
| Influenza PB1 591-599 | A1 |  | V | S | D | G | G | P | N | L | Y |  |  |  |  |  |  |  | C-S-066 |
| Mage-3A1 168-176 | A1 |  | E | V | D | P | I | G | H | L | Y |  |  |  |  |  |  |  | C-S-026 |
| Mage-1 161-169 | A1 |  | E | A | D | P | T | G | H | S | Y |  |  |  |  |  |  |  | C-S-056 |
| Tyrosinase aa 243-251 ana* | A1 |  | K | S | D | I | C | T | D | E | Y |  |  |  |  |  |  |  | C-S-041 |
|  | **HLA-A3** |  | 1 | 2 | 3 | 4 | 5 | 6 | 7 | 8 | 9 |  |  |  |  |  |  |  | Quelle |
| Influenza NP 265-273 | A3 |  | I | L | R | G | S | V | A | H | K |  |  |  |  |  |  |  | C-S-059 |
| Mage-1 aa 96-104 | A3 |  | S | L | F | R | A | V | I | T | K |  |  |  |  |  |  |  | C-S-057 |
| gp100 614-622 | A3 |  | L | I | Y | R | R | R | L | M | K |  |  |  |  |  |  |  | C-S-058 |
|  | **HLA-A24** |  | 1 | 2 | 3 | 4 | 5 | 6 | 7 | 8 | 9 |  |  |  |  |  |  |  |  |
| Tyrosinase 206-214 | A24 |  | A | F | L | P | W | H | R | L | F |  |  |  |  |  |  |  | C-S-067 |
| Mage-1 | A24 |  | N | Y | K | H | C | F | P | E | I |  |  |  |  |  |  |  | C-S-068 |
| gp 100 | A24 |  | V | Y | F | F | L | P | D | H | L |  |  |  |  |  |  |  | C-S-069 |
| Mage-3 195-203 | A24 |  | I | M | P | K | A | G | L | L | I |  |  |  |  |  |  |  | C-S-070 |
|  | **HLA-B44** |  | 1 | 2 | 3 | 4 | 5 | 6 | 7 | 8 | 9 |  |  |  |  |  |  |  |  |
| Tyrosinase 243-251 | B44 |  | S | E | I | W | R | D | I | D | F |  |  |  |  |  |  |  | C-S-071  Bachem H3812 |
| Mage-3 167-176 | B44 |  | M | E | V | D | P | I | G | H | L | Y |  |  |  |  |  |  | C-S-072  Bachem H3686 |
|  |  |  |  |  |  |  |  |  |  |  |  |  |  |  |  |  |  |  |  |
|  | **MHC II** |  | 1 | 2 | 3 | 4 | 5 | 6 | 7 | 8 | 9 | 10 | 11 | 12 | 13 | 14 | 15 | 16 | Quelle |
| Tyrosinase 450-462 ana* | DRB1*0401 |  | S | Y | L | Q | D | S | V | P | D | S | F | Q | D |  |  |  | C-S-060  Bachem H441 |
| gp 100 44-59 | DRB1*0401 |  | W | N | R | Q | L | Y | P | E | W | T | E | A | Q | R | L | D | C-S-061  Bachem H4406 |
| Mage-3 121-134 | DR13 |  | L | L | K | Y | R | A | R | E | P | V | T | K | A | E |  |  | C-S-055 |
| Mage-3 243-258 | DPB1*0401  DPB1*0402 |  | K | K | L | L | T | Q | H | F | V | Q | E | N | Y | L | E | Y | C-S-075 |
