# Supplementary figures and images for: Combining chemotherapy and autologous peptide‐pulsed dendritic cells provides survival benefit in stage IV melanoma patients
Source: J Dtsch Dermatol Ges. 2020 Nov 16;18(11):1270–7. doi: 10.1111/ddg.14334 (PMC7756560; doi:10.1111/ddg.14334)

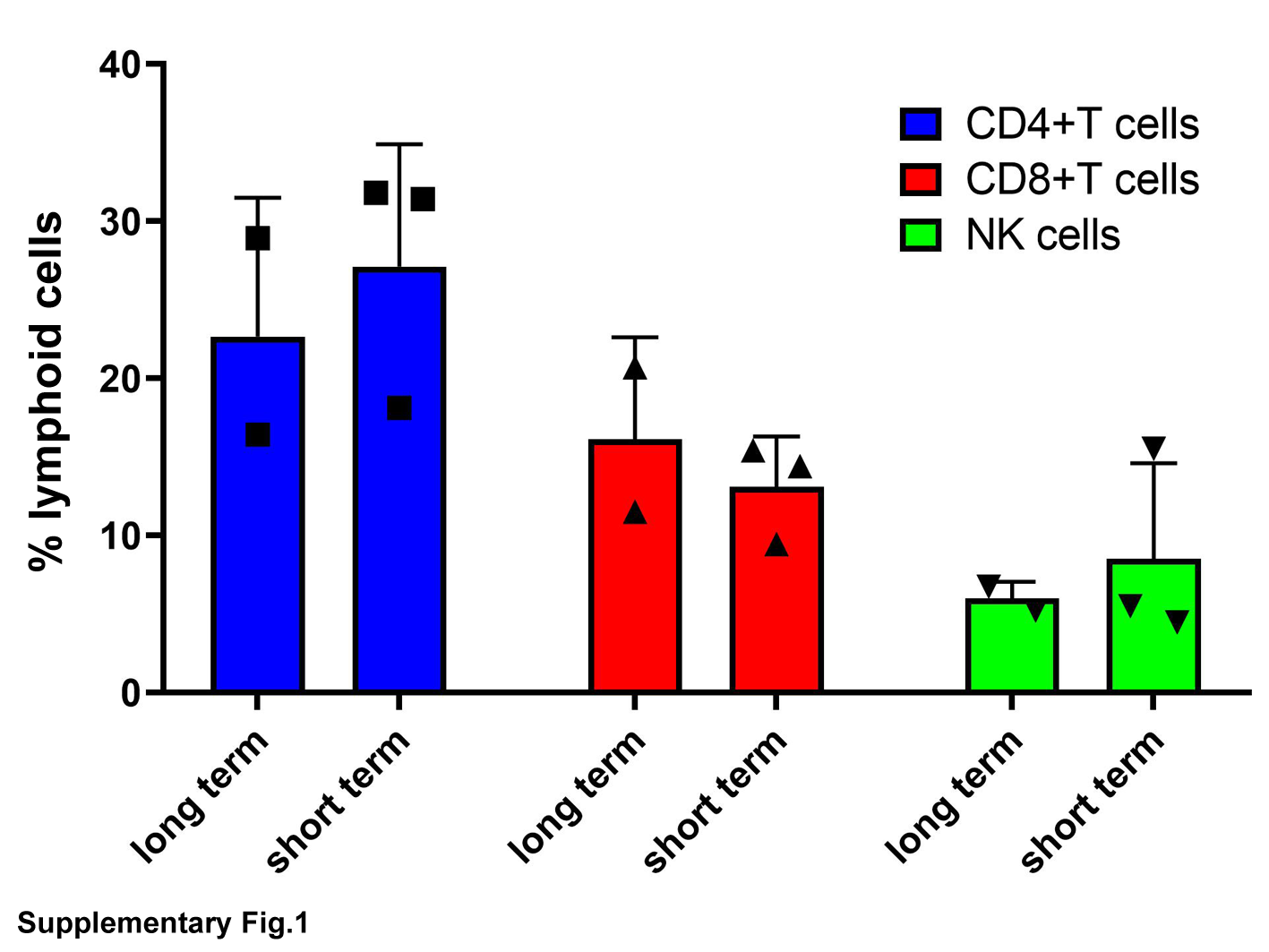

Supplement: Supplementary file 7 — Figure S1 [file DDG-18-1270-s007.tif]

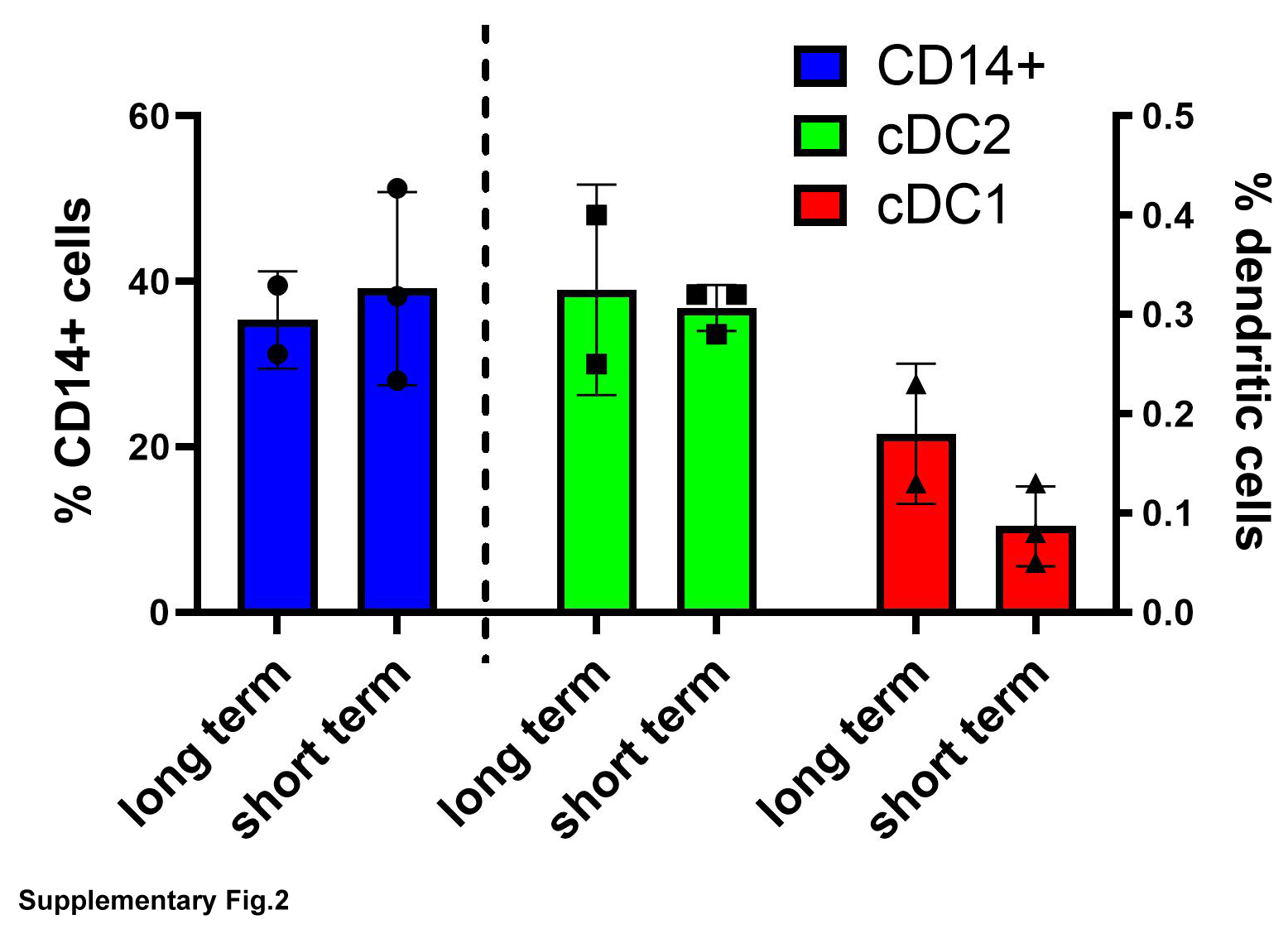

Supplement: Supplementary file 8 — Figure S2 [file DDG-18-1270-s008.tif]
